# Survival inequalities in patients with lung cancer in France: A nationwide cohort study (the TERRITOIRE Study)

**DOI:** 10.1371/journal.pone.0182798

**Published:** 2017-08-25

**Authors:** Christos Chouaïd, Didier Debieuvre, Isabelle Durand-Zaleski, Jérôme Fernandes, Arnaud Scherpereel, Virginie Westeel, Cécile Blein, Anne-Françoise Gaudin, Nicolas Ozan, Soline Leblanc, Alexandre Vainchtock, Pierre Chauvin, François-Emery Cotté, Pierre-Jean Souquet

**Affiliations:** 1 Department of Chest Medicine, Créteil University Hospital, Créteil, France; 2 Department of Chest Medicine, Mulhouse University Hospital, Mulhouse, France; 3 URCEco Île-de-France, Hôtel-Dieu Hospital, Paris, France; 4 Department of Public Health, Henri-Mondor Hospital, Créteil, France; 5 Oc Santé, Montpellier, France; 6 Pulmonary and Thoracic Oncology Department, Lille University Hospital, Lille, France; 7 Department of Chest Medicine, Jean Minjoz University Hospital, Besançon, France; 8 HEVA, Lyon, France; 9 Health Economics and Outcomes Research, Laboratoire Bristol-Myers Squibb, Rueil-Malmaison, France; 10 Sorbonne Universités, UPMC Université Paris 06, INSERM, Institut Pierre Louis d’Epidémiologie et de Santé Publique (IPLESP UMRS 1136), Department of Social Epidemiology, Paris, France; 11 Department of Chest Medicine, Hospices Civils de Lyon, Centre Hospitalier Lyon Sud, Pierre-Bénite, France; Taipei Medical University, TAIWAN

## Abstract

The French healthcare system is a universal healthcare system with no financial barrier to access to health services and cancer drugs. The objective of the study is to investigate associations between, on the one hand, incidence and survival of patients diagnosed with lung cancer in France and, on the other, the socioeconomic deprivation and population density of their municipality of residence. A national, longitudinal analysis using data from the French National Hospital database crossed with the population density of the municipality and a social deprivation index based on census data aggregated at the municipality level. For lung cancer diagnosed at the metastatic stage, one-year and two-year survival was not associated with the population density of the municipality of residence. In contrast, mortality was higher for people living in very deprived, deprived and privileged areas compared to very privileged areas (hazard ratios at two years: 1.19 [1.13–1.25], 1.14 [1.08–1.20] and 1.10 [1.04–1.16] respectively). Similar associations are also observed in patients diagnosed with non-metastatic disease (hazard ratios at two years: 1.21 [1.13–1.30], 1.15 [1.08–1.23] and 1.10 [1.03–1.18] for people living in very deprived, deprived and privileged areas compared to very privileged areas). Despite a universal healthcare coverage, survival inequalities in patients with lung cancer can be observed in France with respect to certain socioeconomic indicators.

## Introduction

With an incidence rate of 41.9 cases per 100,000 individuals in 2012, lung cancer is the fourth most frequent cancer in terms of incident cases in Europe and the leading cause of cancer-related death [1]. In France, the number of incident cases of lung cancer in 2012 was estimated at 40,046 and the number of deaths at 31,343 [1, 2]. Provision of care to patients with lung cancer thus represents a major burden on healthcare systems. Lung cancer incidence has been reported to be higher in areas of socioeconomic deprivation and in urban areas compared to rural areas [3, 4].

Socioeconomic disparities in access to healthcare for patients with lung cancer have been identified in many countries [5–7]. These disparities may translate into differences in survival [5, 7–9], since they create barriers to timely diagnosis and effective management. Several factors may contribute to social gradients in cancer survival, including the patient’s general health status and comorbidities, knowledge about the disease and healthcare-seeking behaviours, the characteristics of the tumour at the time of diagnosis and clinical management. Differences in the management of patients in relation to socioeconomic factors, including the use of surgery in non-small-cell lung cancer patients, have previously been reported in different countries [8, 10]. The identification and reduction of these barriers may improve survival [11] and, for these reasons, research leading to improved understanding of socioeconomic disparities in outcomes and access to care should be a key priority in the management of lung cancer. Several studies in lung cancer have suggested that socio-economic characteristics of patients may influence their outcomes. Survival may also differ according to the distance of the patient from healthcare services, in particular from the nearest cancer centre [12].

The French health care system ensures universal coverage for healthcare through public health insurance funds. In theory, there are no financial barriers to access to health services (especially for the most costly treatments), and sociological and geographical differences should play only a marginal role in outcomes of lung cancer. Nonetheless, data addressing such influences are very limited. The objective of the TERRITOIRE study was to investigate potential associations between socioeconomic markers and lung cancer incidence and survival in France.

## Materials and methods

### Study design

This was a retrospective, longitudinal analysis using data extracted from the French National Hospital database (PMSI; *Programme de Médicalisation des Systèmes d’Information*) relating to all hospitalised patients, crossed with geographically aggregated socioeconomic variables at the lowest local authority level (the *commune*), documented in the databases of the national census of the French national statistics office (INSEE) through individual patient postcodes.

### PMSI database

The PMSI covers all hospitalisations in the public and private sectors involving short-term stays in medical, surgical or obstetric facilities of all hospitalisations in France. The reasons for hospitalisation are coded by ICD-10 diagnosis [13], either as principal diagnoses (PD; the condition for which the patient was hospitalised), related diagnoses (RD; any underlying condition which may have been related to the PD) or as significantly-associated diagnoses (SAD; comorbidities which may affect the course or cost of hospitalisation). Demographic data is limited to age, gender and home address postcode. Patients can be tracked across multiple hospitalisations through a unique anonymous patient identifier, which is conserved until the patient dies. If the patient dies in hospital, this is documented, although the cause of death is not specified.

### Study population

The analysis included all patients with a first hospital stay following diagnosis of lung cancer in 2011 (incident cases). These patients were identified through an ICD-10 code for lung cancer (C34) as PD, RD or SAD for any hospital stay in 2011. Patients were divided into one group diagnosed at the metastatic stage and a second group diagnosed at the non-metastatic stage, since these two groups have a very different prognosis. Metastatic disease was identified from three different sources, namely an ICD-10 code for metastatic disease, hospitalisation in palliative care as a first hospitalisation for lung cancer or administration of chemotherapy for metastatic disease.

Each patient was followed for two years from the initial hospital stay. Patients who died over the two-year follow-up period were censored at the date of death or at the time of the last observation. For each patient, information was documented at the time of initial hospitalisation on gender, age at diagnosis, type of hospital where the patient was first admitted for lung cancer management and the presence of significant chronic comorbidities (hypertension, diabetes mellitus, renal insufficiency, chronic obstructive pulmonary disease, pulmonary insufficiency and other chronic lung diseases) identified as PD, RD or SAD in the patient discharge record. Survival status at one and two years of follow-up was determined. These are the time horizons conventionally used in clinical trials and cancer registries, and thus allow comparison of our data with others. Since the cause of death is not documented, mortality corresponds to all-cause mortality. Death elsewhere than in the hospital is not documented.

The municipality of residence (*commune*) for each patient at the time of the initial hospitalisation was determined from their postcode. Patients whose postcode was not documented in the PMSI database were excluded from the analysis. The *commune* is the lowest tier of local authority in France and generally consists of a single population centre together with any surrounding hamlets or countryside, with a typical area of 10–50 km^2^. There are around 36,000 such municipalities in France. Data were retrieved from the French national statistics office (INSEE) on the sociodemographic make-up of each municipality and used to classify them in terms of population density and social deprivation. Based on the national census data of 2011 and the surface area of the commune, the population density of the municipality was categorised by quartile into four classes: very low, low, high and very high population density. Municipalities were ranked on the basis of a social deprivation index (SDI) determined on the basis of unemployment rate, median household income, the percentage of high school graduates in the adult population and the percentage of blue-collar workers in the active population [14]. This index has been validated previously in the French setting as a tool for evaluating socioeconomic disparities in health at the municipality level. Municipalities were divided by quartile into four classes, corresponding to most deprived, deprived, privileged and most privileged [14]. Population density and SDI are not correlated (Spearman’s rank correlation coefficient: *ρ* = 0.37).

### Statistical analysis

The presentation of the data is descriptive. Continuous data are presented as mean values with standard deviation (SD) or median values, and categorical data as frequency counts and percentages. Incidence and survival rates were calculated overall and by region with reference to the total population of mainland France (or of the region) in 2011. Survival rates by population density of the municipality and by SDI were compared using hazard ratios for four different conditions, namely one-year survival in patients with non-metastatic disease at diagnosis, two-year survival in non-metastatic disease, one-year survival in metastatic disease and two-year survival in metastatic disease. Hazard ratios were adjusted for age, gender and the presence of comorbidities. In a first step, univariate analyses were performed for each of the four conditions to evaluate potential associations between incidence or survival, on the one hand, and each variable of interest on the other. In a second step, variables which showed an association in three or four of the univariate models (p <0.05; χ^2^ test) were entered into corresponding multivariate Cox models for all four of the conditions. All models were multi-level, controlling for the population structure of the sample in order to take into account potential dependence between patients at the municipality level. The models were tested for proportionality of hazard using the Schoenfield partial residuals method [15]. Likelihood ratio testing was used for all tests of significance. All cause-mortality rates were evaluated using Kaplan–Meier survival analysis.

### Ethics

The study was conducted in accordance with International Society for Pharmacoepidemiology (ISPE) Guidelines for Good Pharmacoepidemiology Practices (GPP) and applicable regulatory requirements. Since this was a retrospective study of an anonymised database and had no influence on patient care, ethics committee approval was not required. Approval was obtained from the *Commission Nationale de l'Informatique et des Libertés* (CNIL) with respect to the confidentiality of individual patient data.

### Results

A total of 41,715 incident cases of lung cancer were identified in the PMSI database in 2011. Exploitable postcodes were unavailable in the PMSI database for 306 patients (0.7%) and these patients were excluded from the analyses of population density and social deprivation.

Incidence was significantly higher in men (age-adjusted incidence: 95.3 cases/100 000 individuals) than in women (age-adjusted incidence: 35.1 cases/100 000 individuals) (p<0.0001). Incidence was inversely related to the population density of the municipality (higher rate in municipalities with lower population densities). Such an association was not observed for the SDI, although a trend towards a higher rate in more deprived areas was observed in men (Table 1).

**Table 1:** Incidence of lung cancer in France (2011) as a function of population density and social deprivation.

Age-adjusted incidence (per 100,000 individuals)	Men			Women		
	Estimate	95% CI		Estimate	95% CI	
Population density of municipality	p = 0.0031			p <0.0001		
Very low density	120.5	117.8	123.2	42.5	40.9	44.1
Low density	113.4	110.9	116.0	40.0	38.5	41.5
High density	82.1	80.2	83.9	29.8	28.8	30.9
Very high density	76.7	75.0	78.5	31.7	30.6	32.8
Social deprivation of municipality	p = 0.2280			p = 0.7557		
Most deprived	116.8	114.3	119.4	34.6	33.4	35.9
Deprived	102.9	100.6	105.1	31.8	30.6	32.9
Privileged	105.1	102.5	107.8	34.0	32.7	35.4
Most privileged	96.4	94.1	98.8	35.2	33.9	36.4

The characteristics of these patients are presented in Table 2. At the time of diagnosis, 21,974 patients (52.7%) fulfilled the criteria for metastatic disease. The mean age was 66.4 years. The most frequent comorbidities were pulmonary diseases (mainly COPD), hypertension and diabetes. Renal insufficiency was documented in 4% of patients. Over a third of patients were managed in local hospitals for their first stay. Patients with metastasis at diagnosis were significantly younger and presented a comorbid diagnosed pulmonary disease significantly less frequently.

**Table 2:** Characteristics of patients with a first diagnosis of lung cancer in 2011.

	Patients with a lung cancer diagnosis			
	All patients (N = 41,715)	Metastatic (N = 21,974)	Non metastatic (N = 19,741)	P-value
Mean age ± SD (years)	66.4 ± 11.9	65.9 ± 12.0	66.8 ± 11.9	<0.0001
Gender: male (%)	29,959 (71.8%)	15,659 (71.3%)	14,270 (72.3%)	0.0441
Comorbidities^a^				
Hypertension	10,821 (25.9%)	5544 (25.2%)	5277 (26.7%)	0.0005
Diabetes	4767 (11.4%)	2494 (11.3%)	2273 (11.5%)	0.5984
Renal insufficiency	1736 (4.2%)	887 (4.0%)	849 (4.3%)	0.1775
Pulmonary disease	17,896 (42.9%)	8963 (40.8%)	8933 (45.3%)	<0.0001
COPD	7323 (17.6%)	3262 (14.8%)	4061 (20.6%)	
Respiratory insufficiency	3267 (7.8%)	1718 (7.8%)	1549 (7.8%)	
Other chronic lung diseases	4561 (10.9%)	2024 (9.2%)	2537 (12.9%)	
Hospital type (first stay)				
Local hospital	15,274 (36.6%)	9188 (41.8%)	6086 (30.8%)	<0.0001
University hospital	10,569 (25.3%)	5240 (23.8%)	5329 (27.0%)	
Private hospital	10,330 (24.8%)	4445 (20.2%)	5885 (29.8%)	
Other	5542 (13.3%)	3101 (14.1%)	2441 (12.4%)	
**Population density** of municipality^b^	N = 41,115	N = 21,668	N = 19,447	0.0754
Very **low density**	10,344 (25.2%)	5,552 (25.6%)	4,792 (24.6%)	
**Low density**	10,275 (24.9%)	5,345 (24.7%)	4,930 (25.4%)	
**High density**	10,138 (24.7%)	5,361 (24.7%)	4,777 (24.6%)	
Very **high density**	10,358 (25.2%)	5,410 (25.0%)	4,948 (25.4%)	
Social deprivation of municipality^b^	N = 41,115	N = 21,668	N = 19,447	0.6860
Most deprived	11,302 (27.5%)	5912 (27.3%)	5390 (27.7%)	
Deprived	11,272 (27.4%)	5972 (27.6%)	5300 (27.3%)	
Privileged	8830 (21.5%)	4681 (21.6%)	4149 (21.3%)	
Most privileged	9711 (23.6%)	5103 (23.6%)	4608 (23.7%)	

COPD: chronic obstructive pulmonary disease; SD: standard deviation.

^a^ Patients could have multiple comorbidities and these variables are thus not mutually exclusive.

^b^ Exploitable postcodes were unavailable in the PMSI database for 306 patients with non-metastatic disease and for 294 patients with metastatic disease.

The overall one-year survival rate was 54.2%, significantly higher in patients with non-metastatic disease than in those with metastatic disease (70.8% versus 39.3%, p <0.0001). The overall two-year survival rate was 42%, again significantly higher in patients with non-metastatic disease compared to those with metastatic disease (58% versus 27%, p <0.0001). Median overall survival was 6.6 months [95% CI: 6.4–6.7] for metastatic disease and 18.9 months [18.4–19.1] for non-metastatic disease.

In multivariate analyses, one and two-year survival in patients with non-metastatic disease at diagnosis was higher in younger patients and in women. The presence of renal insufficiency and respiratory insufficiency was associated with poor survival, whereas COPD, and to a lesser extent, hypertension, were associated with improved survival (Table 3). No difference in survival was observed with respect to population density. With respect to social deprivation, survival was significantly lower outside very privileged areas at both one year and two years, being lowest in very deprived areas (21% lower survival at two years), followed by deprived areas (15% lower survival) and privileged areas (10% lower survival). The assumption of proportional hazards was not violated (*p* >0.05; Schoenfeld’s partial residual).

**Table 3:** Variables associated with mortality in patients with non-metastatic lung cancer at diagnosis (Cox model).

COX MODEL	NON-METASTATIC DISEASE AT DIAGNOSIS			
	One-year mortality		Two-year mortality	
	Hazard ratios [95%CI]		Hazard ratios [95%CI]	
	Univariate	Multivariate	Univariate	Multivariate
**Gender**				
Male	1.34	1.27	1.33	1.28
	[1.26; 1.42]***	[1.19; 1.35]***	[1.26; 1.40]***	[1.22; 1.35]***
Female [reference]	1.00	1.00	1.00	1.00
**Age**				
≤ 55 years	0.58	0.59	0.64	0.64
	[0.53; 0.63]***	[0.54; 0.64]***	[0.60; 0.68]***	[0.61; 0.69]***
56 to 65 years	0.65	0.66	0.71	0.71
	[0.61; 0.69]***	[0.62; 0.70]***	[0.67; 0.74]***	[0.67; 0.74]***
≥ 66 years [reference]	1.00	1.00	1.00	1.00
**Comorbidities**				
Hypertension	1.06	0.89	1.01	0.89
	[1.00; 1.13]***	[0.84; 0.95]*	[0.96; 1.06]	[0.84; 0.94]***
Diabetes	1.16	1.03	1.08	0.99
	[1.07; 1.25]***	[0.95; 1.12]	[1.01; 1.15]*	[0.92; 1.06]
Renal insufficiency	1.90	1.58	1.68	1.47
	[1.70; 2.12]***	[1.40; 1.77]***	[1.52; 1.86]***	[1.32; 1.64]***
COPD	0.98	0.82	0.92	0.81
	[0.92; 1.04]	[0.77; 0.88]***	[0.87; 0.97]**	[0.76; 0.86]***
Respiratory insufficiency	2.25	2.28	1.79	1.85
	[2.06; 2.45]***	[2.09; 2.50]***	[1.65; 1.95]***	[1.70; 2.01]***
**Population density**				
Very low density	1.14	1.06	1.09	1.01
	[1.06; 1.23]**	[0.98; 1.15]	[1.02; 1.16]*	[0.94; 1.08]
Low density	0.99	0.95	1.00	0.97
	[0.91; 1.06]	[0.88; 1.03]	[0.94; 1.07]	[0.90; 1.03]
High density	1.05	1.02	1.02	0.99
	[0.97; 1.13]	[0.94; 1.10]	[0.95; 1.09]	[0.93; 1.06]
Very high density	1.00	1.00	1.00	1.00
**Social deprivation index**				
Most deprived	1.27	1.25	1.22	1.21
	[1.18; 1.37]***	[1.16; 1.35]***	[1.14; 1.30]***	[1.13; 1.30]***
Deprived	1.20	1.19	1.16	1.15
	[1.12; 1.30]***	[1.10; 1.29]***	[1.09; 1.24]***	[1.08; 1.23]***
Privileged	1.15	1.14	1.10	1.10
	[1.06; 1.24]**	[1.05; 1.24]*	[1.03; 1.18]**	[1.03; 1.18]*
Most privileged [reference]	1.00	1.00	1.00	1.00

Data are presented as hazard ratios with their 95% confidence intervals [95%CI].

*p < 0.05;

**p < 0.001;

***p < 0.0001.

COPD: chronic obstructive pulmonary disease.

For patients with metastatic disease at diagnosis, one and two-year survival was also higher in younger patients and in women (Table 4). Renal and respiratory insufficiency were again associated with lower survival, whereas no protective effect of COPD or hypertension was observed in this patient group. No association was observed between population density and survival, whereas survival differences were again observed with respect to social deprivation. At Year 2, survival was reduced by 19% in very deprived areas, 14% in deprived areas and 10% in privileged areas compared with very privileged areas.

**Table 4:** Variables associated with mortality in patients with metastatic lung cancer at diagnosis (Cox model).

COX MODEL	METASTATIC DISEASE AT DIAGNOSIS			
	One-year mortality		Two-year mortality	
	Hazard ratios [95%CI]		Hazard ratios [95%CI]	
	Univariate	Multivariate	Univariate	Multivariate
**Gender**				
Male	1.25	1.21	1.22	1.18
	[1.20; 1.30]***	[1.16; 1.26]***	[1.18; 1.26]***	[1.14; 1.23]***
Female (reference.)	1.00	1.00	1.00	1.00
**Age (inclusion)**				
≤ 55 years	0.65	0.69	0.70	0.73
	[0.63; 0.68]***	[0.66; 0.72]***	[0.67; 0.73]***	[0.70; 0.76]***
56 to 65 years	0.71	0.73	0.74	0.77
	[0.68; 0.73]***	[0.70; 0.76]***	[0.72; 0.77]***	[0.74; 0.80]***
≥ 66 years (reference)	1.00	1.00	1.00	1.00
**Comorbidities**				
Hypertension	1.15	0.96	1.13	0.96
	[1.11; 1.20]***	[0.92; 1.00]	[1.09; 1.17]***	[0.92; 1.00]
Diabetes	1.22	1.04	1.20	1.05
	[1.16; 1.29]***	[0.98; 1.11]	[1.15; 1.27]***	[1.00; 1.11]
Renal insufficiency	2.13	1.72	2.02	1.68
	[1.95; 2.33]***	[1.56; 1.90]***	[1.85; 2.21]***	[1.52; 1.85]***
COPD	1.19	1.00	1.17	1.00
	[1.14; 1.25]***	[0.95; 1.05]	[1.11; 1.22]***	[0.95; 1.05]
Respiratory insufficiency	2.40	2.24	2.23	2.08
	[2.24; 2.57]***	[2.08; 2.40]***	[2.07; 2.39]***	[1.93; 2.24]***
**Population density**				
Very low density	1.02	0.96	1.01	0.95
	[0.96; 1.07]	[0.90; 1.02]	[0.96; 1.06]	[0.90; 1.01]
Low density	0.98	0.97	1.00	0.99
	[0.93; 1.04]	[0.92; 1.03]	[0.95; 1.05]	[0.94; 1.05]
High density	0.98	0.96	0.98	0.95
	[0.93; 1.04]	[0.91; 1.02]	[0.93; 1.03]	[0.90; 1.00]
Very high density	1.00	1.00	1.00	1.00
**Social deprivation index**				
Most deprived	1.17	1.19	1.16	1.19
	[1.11; 1.23]***	[1.13; 1.26]***	[1.11; 1.21]***	[1.13; 1.25]***
Deprived	1.09	1.13	1.10	1.14
	[1.04; 1.15]**	[1.07; 1.20]***	[1.05; 1.15]***	[1.08; 1.20]***
Privileged	1.09	1.11	1.08	1.10
	[1.03; 1.15]*	[1.04; 1.18]*	[1.03; 1.13]***	[1.04; 1.16]*
Most privileged (reference)	1.00	1.00	1.00	1.00

Data are presented as hazard ratios with their 95% confidence intervals [95%CI].

*p < 0.05;

**p < 0.001;

***p < 0.0001.

COPD: chronic obstructive pulmonary disease.

## Discussion

In this nationwide cohort study, we observed significant socio-geographic inequalities in the incidence and survival of patients with lung cancer in France. Other than demographic and clinical variables that have been reported previously to be associated with survival, such as sex, age, disease stage and comorbidities, our study also demonstrated differences in survival related to the level of social deprivation of the *commune* where the patient lived. In particular, one- and two- year survival was significantly lower in patients living in socially deprived areas compared to very privileged ones. These differences were observed both for cancers that were diagnosed at the metastatic stage and for those diagnosed at the non-metastatic stage. With respect to the population density of the municipality, no such association with survival was observed.

As previously demonstrated elsewhere [16], stage at diagnosis, age and gender were strongly predictive of one and two-year survival for lung cancer. In our analysis, the overall one-year survival rate was 54%, significantly higher in patients with non-metastatic disease compared to metastatic disease (71% versus 39%). Whatever the disease stage at diagnosis, survival rates were higher in women than in men. Such a difference has also been observed in a study performed in England, in which the one-year mortality rate for women with lung cancer was 13% lower than in men [16]. Similarly, data from a recent French patient registry reported higher mortality rates in men (56.6%) than in women (50.9%) [17]. Finally, older age was associated with lower survival, as previously described [16]. Moreover, higher mortality rates have been reported for rural and suburban residents with lung cancer in the United States compared to their urban counterparts [18]. The reasons for such adverse survival outcomes for lung cancer could be poorer access to specialist physicians, and in particular oncologists and pulmonologists.

Less data are available on the relationship between comorbidities and survival in lung cancer. Our study shows that, regardless of the stage of the disease at diagnosis, comorbid kidney failure and chronic respiratory impairment were negatively associated with survival whereas the presence of COPD appeared to be protective in non-metastatic disease. It could be postulated that such a protective effect could be due to the fact that patients with COPD are likely to be already followed by a pulmonologist before their cancer develops, thus increasing the probability of early diagnosis and management by a specialist. A Danish study of 13,045 patients with lung cancer [6] recently demonstrated a strong association between the Charlson Comorbidity Index score and survival, with an over twofold decrease in survival in patients with more than three comorbidities compared with those without comorbidities. Moreover, a recent systematic review of a number of population-based cohort studies in which the Charlson Comorbidity Index had been used to measure comorbidity found that, although patients with comorbid conditions had less advanced lung cancer, they were nevertheless less likely to receive treatments recommended in practice guidelines [19].

Survival inequalities in patients with lung cancer have been the subject of numerous recent studies and our findings are in line with those of several other population based studies of the impact of social inequalities on survival of patients with lung cancer, both in universal [3,4,6] and in non-universal healthcare systems [20]. Some of these studies have been conducted at the individual patient level and others at the community level. For example, in patients with advanced-stage disease in Denmark, the relative risk of death was 1.12 (95% CI 1.05–1.19) for patients with low income compared to those with high income [6], with the differences in mortality risk being greatest in the first six months after diagnosis. Another individual patient study conducted in the UK found that social deprivation was associated with early death (within 90 days of diagnosis) from lung cancer [21]. More frequently, published studies have, like ours, been performed at the community level (ecological studies). These studies have used proxy markers of social deprivation based on an aggregate measure for where the patient lives. Notably, a previous French study reported data on patients with lung cancer in a patient registry in the Franche Comté region in the east of the country in 2001 [22]. This study compared survival between patients living in municipalities classified using a composite measure of rurality, including indices of population density, structure and evolution (*eg* migration), social deprivation (*eg* education and employment status) and land use. Poorer survival was observed in more rural areas [22]. Studies in Canada [23] and the USA [20, 24] have shown that area-based socioeconomic status (defined by census tracts, electoral enumeration areas or postcodes) was independently associated with survival even after adjustment for surgery, race, marital status or age. In a study of 76,086 lung cancer patients in the USA, adjustment for stage, treatment and comorbidity accounted for part of the association between postcode-based socioeconomic status and survival, although patients living in low-status areas still had a slightly worse prognosis than affluent patients (HR 1.05; 95% CI 1.02–1.09) [24]. A study in England showed that relative survival decreased with greater income deprivation, with a difference of 2.6% between the least and most deprived areas (defined by postcodes) [5]. In an analysis of 215,000 patients in a national US Cancer Data Base, living in lower-income neighbourhoods (defined by postcodes), with a lower proportion of high school graduates, was associated with increased 30-day postoperative mortality after lung cancer resection [25]. Nonetheless, caution should be exercised in inferring health patterns at the individual level from these community studies. The finding that mean survival differs between municipalities with different levels of social deprivation may not necessarily indicate that poorer individuals with lung cancer living in these communities will have a lower likelihood of survival than richer individuals (an example of the ecological fallacy) due to the presence of other individual risk factors not taken into account in our study, such as environmental pollution.

One of the difficulties in interpreting such studies lies in the non-uniform definition of social deprivation used, notably since individual components of deprivation, such as low education level, may also be associated with risk factors for lung cancer, such as smoking. Of interest in this respect is a recent French nationwide observational study which analysed potential links between vulnerable social status, identified using a validated questionnaire (EPICES), exposure to lung cancer risk factors and access to healthcare, through telephone interviews of a representative sample of 1603 subjects [26]. The socially vulnerable population presented more risk factors for lung cancer than non-vulnerable individuals, notably a higher BMI, a higher active smoking rate with a heavier and longer-lasting tobacco consumption and a lower level of physical activity. They also presented on average more comorbidities. Access to healthcare, however, was not discriminatory, since vulnerable individuals declared consulting a general practitioner or an oncologist more often than non-vulnerable individuals (5.4 vs. 3.7 and 6.7 vs. 2.5 consultations in the previous 12 months, respectively; *p* ≤0.01).

This type of study does not allow the causes of the inequity in survival according to socio-geographic disparity to be addressed directly. However, a number of hypotheses may be put forward which may be useful to orientate future research. For example, a study in Scotland has demonstrated that cancer patients living in rural areas have lower care expectations than their urban counterparts [27], which may lead to delays in seeking treatment. In contrast, better-educated individuals may have higher expectations and be more proactive in seeking medical support [5]. Distance to treatment centres has also been proposed to contribute to poorer survival in patients living in rural areas or small towns of England [28]. It is also possible that individuals living in low socio-economic groups may have poorer lung health in general, with higher smoking rates [26, 29] and exposure to industrial or environmental pollutants, which may lead to early symptoms of lung cancer going unrecognised. For example a recent study suggested that higher *per capita* lung cancer mortality in rural areas of the United States was driven by higher rural smoking rates [30]. In addition, individuals in areas of social deprivation may be more unhealthy in general. It is important to identify and address any such barriers to healthcare. Potential ways to reduce these inequalities could include actions to encourage general practitioners to ensure that smokers and people exposed to environmental pollution undergo regular chest X-rays or funding of mobile ‘lung clinics’ to visit deprived areas.

This study was performed using data from the PMSI database. This choice was based on the fact that this is an exhaustive data on patients with lung cancer at a national level. Although regional or local cancer patient registries exist in France, there is no specific national registry for lung cancer with longitudinal data. Since the PMSI database has been used to attribute hospital funding on a pay by activity basis since 2005, the quality and exhaustiveness of data coding has improved considerably, and a recent comparison of standardised incidence ratios for cancer determined from the PMSI and from local cancer registries has shown that the two sources provide very similar estimates [31]. Although the PMSI database contains data on all patients in France hospitalised with lung cancer and on their treatments, the reasons for, or results of, any tests or procedures performed are not documented. The information on treatment would be interesting to explore in future analyses to investigate whether geographic variables have any impact on treatment.

The strengths of our study include the population-based approach, with a cohort of all lung cancer patients managed in France in one year. Nonetheless, the use of this data source presents certain drawbacks. Firstly, it is not possible to characterise the tumour with respect to histological type or detailed stage of cancer (for example with the TNM Classification of Malignant Tumours) in the PMSI database, since this information is not captured in the DRG code used to define the hospital stay. This lack of access to disease and treatment variables is a major limitation of the study, since such variables may influence the association between geographic variables and survival. However, the proportion of patients with metastatic disease at diagnosis in our study (53%) is close to that observed in a recent nationwide survey of lung cancer in France based on patient records which used TMN codes (58% in 2010) [32]. Although undocumented tumour characteristics may influence survival, it may be assumed that the distribution of cancer types will not differ according to the sociodemographic characteristics of the municipality of residence. However, we are not able to validate this assumption.

Secondly, our individual-level demographic variables were limited to gender and age and we lack information on important variables such as smoking status. Thirdly, it is also possible that a competing risk of death from causes other than lung cancer played a part in the observed overall mortality rates. Certain competing causes of death such as injury may also be associated with social deprivation, and some contamination from such causes cannot be excluded. Nonetheless, we have assumed that the major contribution to death in our sample will be lung cancer itself. Some imprecision also arises since certain patients with lung cancer do not die in hospital and thus will not be captured in the PMSI database. However, an exhaustive survey of the relationship between place and cause of death in France has demonstrated that around 80% of patients who die from lung cancer die in hospital [33]. In addition, censure of the data at the time of the last observation limits any potential bias in survival estimation due to potential deaths outside hospital. Finally, we could only estimate socioeconomic variables at the level of the municipality of residence of the patient, which is only a proxy marker of individual socioeconomic status; and may also mask diversity in socioeconomic status between different districts of the same municipality.

The originality of our study lies in the use of a nationwide comprehensive health insurance database to identify cases. Firstly, this has the advantage of ensuring quasi-exhaustiveness of case identification, and thus eliminating the problem of sampling bias which is common to registry studies. Secondly, comparisons are possible with other diseases identifiable in the same database to detect potential disease-specific barriers to health equality. Finally, similar approaches can be taken in other countries where nationwide comprehensive health insurance databases exist, such as Scandinavia, in order to identify country-specific differences in health equality.

## Conclusion

Although France is a highly egalitarian country with a free, universal healthcare system, this study found survival inequalities in patients with lung cancer related to residential socioeconomic indicators. It will be important to understand these associations in order to propose and implement strategies to ensure territorial equity in opportunity for patients diagnosed with lung cancer.
